# Communal breeding by women is associated with lower investment from husbands

**DOI:** 10.1017/ehs.2022.47

**Published:** 2022-10-20

**Authors:** Qiao-Qiao He, Jun-Wen Rui, Li Zhang, Yi Tao, Jia-Jia Wu, Ruth Mace, Ting Ji

**Affiliations:** 1 College of Life Science, Shenyang Normal University, Shenyang, Liaoning 110034, China; 2 Key Laboratory of Animal Ecology and Conservation Biology, Institute of Zoology, Chinese Academy of Sciences, Beijing 100101, China; 3 Life Science, Lanzhou University, Tianshui Rd, Chengguan Qu, Lanzhou, Gansu Province, 730000, China; 4 Department of Anthropology, University College London, London WC1H 0BW, UK

**Keywords:** Communal breeding, matrilineal puzzle, kin selection, Mosuo, matrilineal society

## Abstract

According to Hamilton's rule, matrilineal-biased investment restrains men in matrilineal societies from maximising their inclusive fitness (the ‘matrilineal puzzle'). A recent hypothesis argues that when women breed communally and share household resources, a man should help his sisters' household, rather than his wife's household, as investment to the later but not the former would be diluted by other unrelated members (Wu et al., 2013). According to this hypothesis, a man is less likely to help on his wife's farm when there are more women reproducing in the wife's household, because on average he would be less related to his wife's household. We used a farm-work observational dataset, that we collected in the matrilineal Mosuo in southwest China, to test this hypothesis. As predicted, high levels of communal breeding by women in his wife's households do predict less effort spent by men on their wife's farm, and communal breeding in men's natal households do not affect whether men help on their natal farms. Thus, communal breeding by women dilutes the inclusive fitness benefits men receive from investment to their wife and children, and may drive the evolution of matrilineal-biased investment by men. These results can help solve the ‘matrilineal puzzle'.

## Graphical abstract

**Figure:**
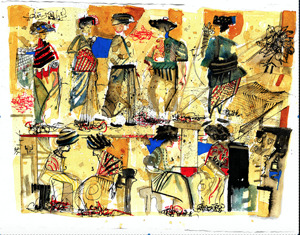

**Social media summary:** High levels of communal breeding by women in the wife's household predict less effort spent by men on their wife's farm.

## 1. Introduction

Whether or not men help raise their offspring can be a source of conflict between spouses. In matrilineal societies, the lack of help from fathers is often extreme, as men are more focused on helping their natal family, or themselves. This ‘matrilineal puzzle' involves the evolutionary puzzle of why men in matrilineal societies invest in their sisters' offspring more than their own (Hartung, 1985). According to Hamilton's rule (Hamilton, 1964), a man should help his own offspring more than sister's offspring, as he is more related to the former than latter (Greene, 1978). However, in matrilineal societies, which used to comprise about 17% of human societies (Murdock, 1967), men favour matrilineal-biased investment, such as transferring their property to their sister's son (Schneider & Gough, 1961) or other forms of investment (Starkweather & Keith, 2019). In some matrilineal groups, such as the matrilineal Mosuo of southwest China where men do not reside with their wife or children (duolocal residences, ‘zouhun' or ‘visiting marriages', are discussed in more detail below), men are not obligated to take care of their own offspring, but have to look after their sister's children.

Previously, we modelled this problem by looking at men's optimal allocation of effort among three realms – their sister's farm, their wife's farm and activities enhancing extra-pair reproductive success. We argued that, if resources are shared within the household, the average relatedness of a man with the whole household will be more important than that with a specific individual (Wu et al., 2013). Thus, when sisters pool their children and share care and provisioning (communal breeding; Lukas & Clutton-Brock, 2012), a man should help the household that he is more related to – which is his sisters' household, rather than his wife's household – as fitness benefits of investment in his wife's household will be diluted by helping to feed the unrelated children of his wife's matrilineal kin (Wu et al., 2013). The hypothesis assumes that household resources can be used by all household members, and especially reproducing women in the household will use a lot of household resources to reproduce (investing in reproducing and rearing children). This assumption is applicable to the matrilineal Mosuo, where sharing of household resources has been well documented (Cai, 2001; Ji et al., 2013; Mattison, 2011; Shih, 2009; Yan & Liu, 2009; Zhan et al., 1980). It also assumes that the benefits a household derives rise towards an asymptote as the amount of investment increases (i.e. as more fields of a household are planted or harvested, additional labour become less useful), and also assumes the levels of communal breeding in the sister's and the wife's households are the same. So only part of his investment will be used by his wife and offspring and bring him fitness benefits, and others will be used by his wife's co-resident relatives, who are unrelated to him. In contrast, all his investment to his sister's household will be used by his relatives and will increase his inclusive fitness. Higher levels of communal breeding are associated with lower average relatedness of a man to his wife's household, whilst communal breeding of men's female kin will not reduce men's average relatedness to their natal households, or the inclusive fitness return of their investment in their natal household. Wu et al. (2013) predicted that, when there were two or more women communally breeding in each household, men could maximise their inclusive fitness by devoting more to their sister's farm than to their wife's farm. Furthermore, high levels of paternity uncertainty were predicted to contribute little to a man's working on his sister's farm vs. his wife's farm, but could reduce men's overall work rates. If average paternity uncertainty is high, a man may get more fitness from pursuing more mates.

Previous hypotheses to solve the matrilineal puzzle emphasised either the man's paternity uncertainty (Alexander, 1974; Anderson, 2006; Greene, 1978; Trivers, 1972) or polygyny (Fortunato, 2012). It is argued that matrilineal investment can evolve only under unrealistically low paternity certainty (0.268–0.33, Greene, 1978; Alexander, 1974, 1977; Kurland 1979) or that matrilineal inheritance is only likely to be a stable evolutionary strategy when men are polygynous and have multiple wives and brothers-in-law, and they will benefit from the investment of multiple brothers-in-law in their wife's offspring (Fortunato, 2012). Neither hypothesis predicts effects of communal breeding by women on a man's help of his wife's household.

In contrast, Wu et al. (2013) predicted that high levels of communal breeding will decrease a man's help on his wife's farm, as it is negatively related to a man's average relatedness to his wife's household, which reduces the fitness benefits of effort invested in his wife's household. Here we test empirically whether the probability of a man helping on his wife's farm decreases with the level of communal breeding in their wife's household. We use our data on who was observed working on which farm to test three predictions derived from this hypothesis (Wu et al., 2013).

First, we test the prediction that the number of reproducing women in a woman's household is negatively correlated to her husband's relatedness to her household (prediction 1). Then we explore whether levels of communal breeding in a woman's household affect the probability of her husband's help on her farm. We predict that a zouhun man will be less likely to help on his wife's farm if there are more reproductive-age or reproducing women in her household (prediction 2).

Finally, it is possible that reduced investment of men is due to the asymptotic nature of the benefits of help on any one farm, not to the decreased average relatedness or associated fitness benefits. If so, high levels of communal breeding of women in men's natal household will also reduce men's investment in their natal farms. Nonetheless, high levels of communal breeding by female kin in a man's natal household will not affect much of his average relatedness or fitness benefits from investing in his natal household. Thus, we predict that a large number of reproducing/reproductive-age women will reduce a man's labour effort to his natal household less than that to his wife's household (prediction 3).

## 2. Material and methods

### (a) Study population

Our study population is the matrilineal Mosuo (also called Moso or Na), inhabiting a geographically constrained habitat surrounded by hills that are not suitable for farming. They mainly reside near and around Lugu Lake on the border of Sichuan and Yunnan provinces in southwestern China. The matrilineal Mosuo are one of the rare populations applying duolocal residence and ‘visiting marriage' (also called ‘zouhun' in Mandarin, or ‘sese' in Na; Shih, 2000; Walsh, 2004). There is also a group of patrilineal Mosuo, located in distinct geographical regions with steeper mountains (Mattison et al., 2016; Shih, 1993, 2009). In total, the Mosuo have a population of about 40,000 (Walsh, 2004). The present study focused on a matrilineal subpopulation of Mosuo.

Traditionally, matrilineal Mosuo women and men live with matrilineal kin, and usually do not disperse in their lifetime. The husband visits the wife at night, and returns to his natal household in the day (Shih, 1993), thus men do not reside with their own offspring (He et al., 2016; Ji et al., 2013; Thomas et al., 2018; Wu et al., 2013). The matrilineal Mosuo men are attributed more responsibilities to their nephews/nieces than their own children, but the importance of fathers may be increasing now (Ji et al., 2016; Mattison et al., 2014; Xiao et al., 2022). In the Mosuo in our study area, about half of the adults practise visiting marriage and duolocal residence (He et al., 2016; Ji et al., 2016; Wu et al., 2013). The levels of polygamy might have been higher in the past (Cai, 2001; He et al., 2016; Wang & Zhan, 2009; Yan & Liu, 2009), but nowadays reported rates of multiple paternities are low (He et al., 2016; Ji et al., 2013). In most circumstances, paternity is acknowledged by the father's family (Shih, 2000; Wu et al., 2013). There might be some relationships not reported by our informants though, especially those childless ones. For simplicity, we use the terms ‘husband' and ‘wife' to refer to men and women identified as zouhun in the household surveys.

Agriculture is the main means of subsistence for the matrilineal Mosuo, although tourism has become more important since the 1980s (Mattison, 2010). In the planting or harvest season, farm work is highly communal and households usually get help on the farm from other households (Thomas et al., 2018; Wang & Zhan, 2009).

### (b) Data collection and analysis

We conducted demographic and socioeconomic surveys in all five matrilineal Mosuo villages in Lugu Lake Town, Sichuan Province, during 2012. Ethical clearance was given by the Chinese Academy of Sciences, Beijing (ref. IOZ12015) and UCL research ethics (ref. 0449002). One adult was interviewed about information for all household members, including name, age, sex, ethnic group, marital status, the names of spouses and parents, etc. Locations were also recorded for each household. Based on this dataset, we calculated the number of reproductive-age women of 15–50 years old co-residing in each household, women with dependents of 14 years or less (reproducing women) and the number of children of all ages fathered by men in their wife's household. We also calculated relatedness between each pair of individuals, based on reported parentage to assemble genealogies of the study households (Wu et al., 2013; Mace et al., 2018; Thomas et al., 2018). Average relatedness of a man to his wife's household was calculated based on this relatedness matrix, and equalled the mean of his relatedness to all members of his wife's household.

During the planting seasons of the years 2011 and 2012 and the harvest season of year 2012, we did spot observations during daylight on who was working on the land belonging to a random sample of farms. Labour demand was high during these periods; all planting or harvesting in this area is complete in 15–20 days. Locations were randomly sampled within the study area, and the coverage was unbiased, although incomplete. We recorded all of the personal information of everyone seen working in a field, including their name, sex, ethnic group, age, animal sign of the year of birth and relationship with the landowner. All analyses were based on a subsample of 334 zouhun Mosuo women, living in 209 landowner households (defined as households for which farm work had been observed on their land in the investigation). We also did some analyses with 261 zouhun Mosuo men whose natal farms had been observed in the investigation. Note that these women or men were not necessarily seen working on any farm. For each woman, only one husband was reported at that time.

**Figure 1.**
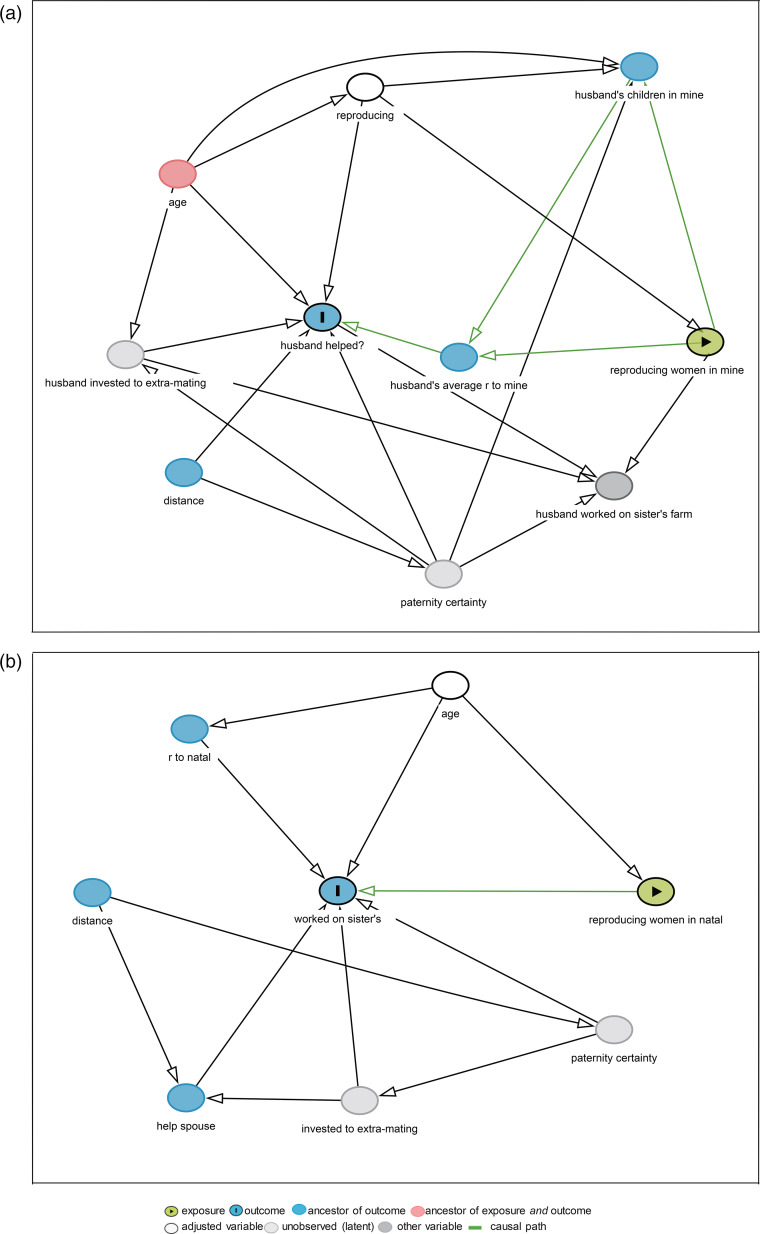
Directed acyclic graph: (a) with number of reproducing/reproductive-age women in women's households (reproducing women in mine) as exposure, and husband's help on women's farms (husband helped?) as outcome; (b) with number of reproducing women in men's natal households (reproducing women in natal) as exposures, and his help on natal farms (worked on sister's) as outcome. Husband's average *r* to mine refers to husband's average relatedness to a woman's household. Husband's children in mine refers to husband's children of all ages in women's households. *R* to natal refers to a man's average relatedness to his natal household.

We used directed acyclic graphs (DAGs) and Pearl's back-door criterion (Pearl, 2000), as implemented in DAGitty (Textor & Hardt, 2011; Textor & Liśkiewicz, 2011), to select minimal sufficient adjustment sets (MSASs) of variables that would enable the distinguishing of an unconfounded effect of communal breeding of women on the husband's help on a woman's farm. We illustrated a DAG to present predictions 1 and 2 (Figure 1a), suggesting that high levels of communal breeding in a woman's household reduced the husband's investment by decreasing average relatedness (and associated fitness benefits). The confounders included age, husband's investment in the sister's farm or extra-pair mating (unobserved variable), average relatedness, husband's children in the focal woman's household (Wu et al., 2013) and distance (Thomas et al., 2018). Paternity certainty was an unobserved variable (Alexander, 1974; Anderson, 2006; Greene, 1978; Trivers, 1972; Wu et al., 2013). Details of the DAG are described in Supplementary Material S1. Variables in the selected MSAS closed all biasing paths, left the causal path open (Thomas et al., 2018; Wu et al., 2013) and included a dichotomous variable on whether a woman was reproducing (1= yes, 0 = no, defined as she had children under the age of 15). Figure 1a shows that after adjusted ‘reproducing', there are no biasing paths (pink line) in the DAG.

We fitted generalised estimating equations (GEEs) to test the effect of communal breeding on whether a man helped his wife's household (1= helped; 0 = not helped). GEEs were specified with an exchangeable correlation structure and observations were clustered on women's households. The final models adjusted reproducing by only including reproducing zouhun women (Figure 1a). Although reproducing women use most household resources to rear their children, reproductive-age women are all potential competitors for household resources used for reproduction (Ji et al., 2013). Thus, we measured the communal breeding levels of reproducing women's households with two alternative variables, number of reproducing women and reproductive-age women in their households.

We also analysed how the number of reproducing/reproductive-age women in a zouhun man's natal household affected his investment in his natal farms. We illustrated a DAG to present prediction 3, with the number of reproducing/reproductive-age women in a man's natal household as the exposure (Figure 1b). The outcome was whether a man helped his natal farms (1= helped; 0 = not helped). We selected a MSAS including age. GEEs were fitted with all variables in the MSAS, and observations were clustered on men's natal households.

DAGs were illustrated from the website of DAGitty (http://www.dagitty.net/) and minimal sufficient adjustment sets were also estimated (Textor & Hardt, 2011; Textor & Liśkiewicz, 2011). All the other analyses were done in R v4.2.0 (R Core Team, 2022), and figures were built with ‘gglot2' package (Wickham, 2016) and the predict function. Relatedness between each pair of individuals was calculated using ‘AGHmatrix' package (Amadeu et al., 2016). Correlation matrix was done using ‘Hmisc' package (Harrell, 2022). GEEs were done with ‘geepack' package (Halekoh et al., 2006; Yan, 2002; Yan & Fine, 2004).

## 3. Results

We observed farm work on 525 farms belonging to 388 households, of which 209 households contained 334 Mosuo zouhun women (Table 1; He et al., 2016; Ji et al., 2013; Wu et al., 2013) at that time. A total of 24.5% women's households had been helped by their husbands (Table 1).

**Table 1.** Descriptive statistics of 334 Mosuo women and their households. Reproducing women have dependants under age 15, and others are defined as non-reproducing

	Households	Women
Zouhun women	209	334
Non-reproducing		139
Reproducing		195
Mean age in years ± SD (range)		39.62 ± 13.41 (16–88)
Non-reproducing women		49.40 ± 14.84 (16–88)
Reproducing women		32.66 ± 5.93 (20–46)
Number of women whose households are ever helped by their husband	74	82
Number of women whose households are never helped by their husband	173	252
Mean number of reproducing women per household ± SD (range)		1.39 ± 0.98 (0–5)
Non-reproducing women		0.95 ± 0.97 (0–5)
Reproducing women		1.71 ± 0.85 (1–5)
Households ever helped		1.48 ± 0.64 (1–4)
Households never helped		1.79 ± 0.90 (1–5)
Mean number of reproductive-age women per household ± SD (range)		2.87 ± 1.46 (0–8)
Non-reproducing women		3.08 ± 1.62 (0–8)
Reproducing women		2.73 ± 1.32 (1–7)
Households ever helped		2.39 ± 1.21 (1–7)
Households never helped		2.85 ± 1.34 (1–7)
Husband's average relatedness		0.13 ± 0.08 (0–0.38)
Non-reproducing women		0.15 ± 0.10 (0–0.38)
Reproducing women		0.11 ± 0.06 (0–0.30)
Households ever helped		0.13 ± 0.06 (0.04–0.30)
Households never helped		0.10 ± 0.06 (0–0.30)

### (a) Communal breeding and husband's average relatedness

**Figure 2.**
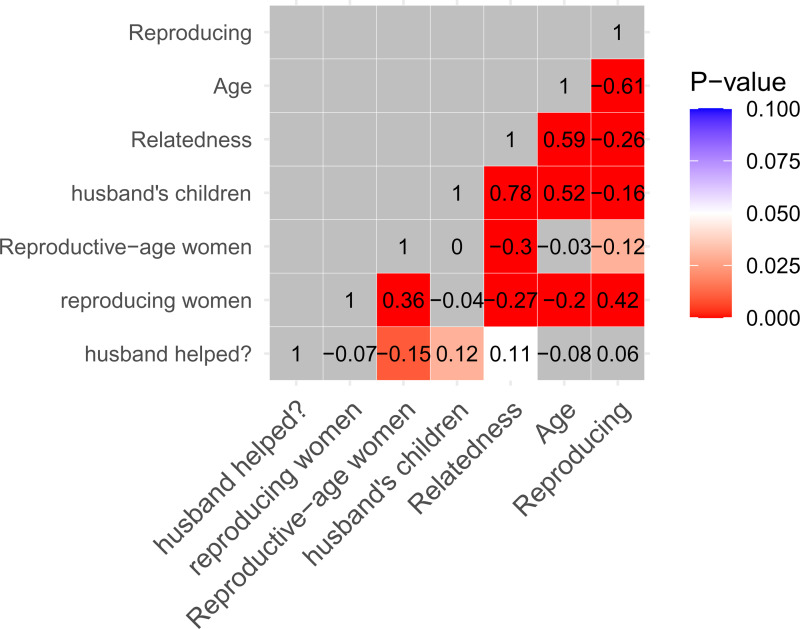
Bivariate correlations between some predictor variables and the dependent variable (husband helped?). Numbers within the cells are Spearman's correlation coefficients. Red cells are statistically significant (*p* < 0.05), with darker shades as *p* approaches zero; blue cells are marginally significant; and grey cells are not statistically significant. Age refers to zouhun women's age in years. Relatedness refers to husband's average relatedness to a woman's household. Husband's children includes children (of all ages) fathered by a man in his wife's household.

On average, there were 2.87 reproductive-age women (range 0–8, SD = 1.46, Table 1) and 1.39 reproducing women per household (range 0–5, SD = 0.98, Table 1). Mean husbands' average relatedness to women's households was 0.13 (*n* = 334 Mosuo women, range 0–0.38, SD = 0.08, Table 1), and relatedness between husbands and reproducing women's households was even lower (mean = 0.11, range 0–0.30, SD = 0.06). Husbands' average relatedness to a woman's household, was strongly associated with the number of his children in her household, and was negatively correlated to the number of reproductive-age and reproducing women living in reproducing women's households, supporting prediction 1 (Figure 2). Moreover, the negative relationship between reproducing women in a household and the husband's average relatedness to it was bigger than that for reproductive-age women.

**Figure 3.**
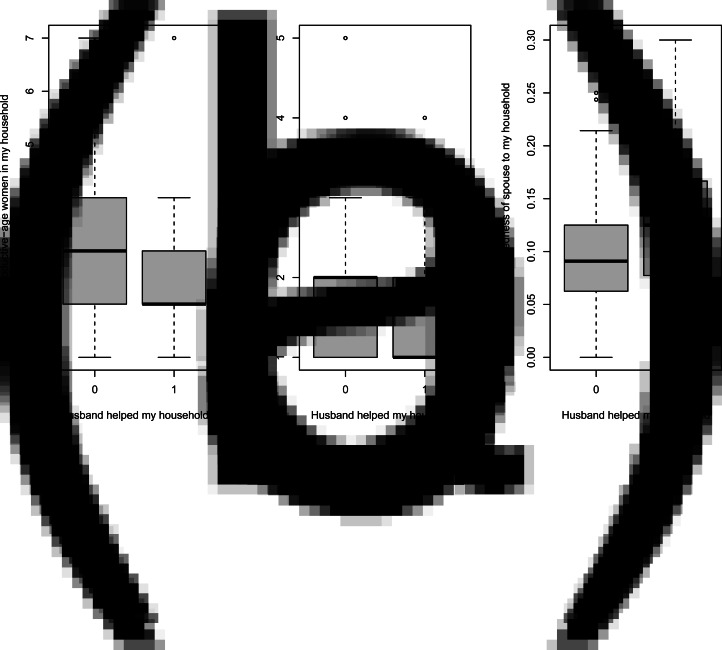
Help from husbands of reproducing Mosuo women. In a visiting marriage, a woman's household was less likely to be helped by her spouse, (a) where more reproductive-age women lived (Wilcoxon rank sum test, *w* = 4504.5, *p* = 0.02), (b) where more reproducing women lived (Wilcoxon rank sum test, *w* = 4405, *p* = 0.03, or (c) when her household was on average less related to her spouse (Wilcoxon rank sum test, *w* = 2845, *p* = 0.01).

Fewer reproductive-age or reproducing women lived in households helped by reproducing Mosuo women's husbands than those not helped (Figure 2; *n* = 195, excluding 140 women without dependents; see also Figure 3a and b, respectively). Similarly, male spouses were more closely related to households which were helped by woman's husbands than those not helped (Figures 2 and 3c).

### (b) Help on wife's farm

**Figure 4.**
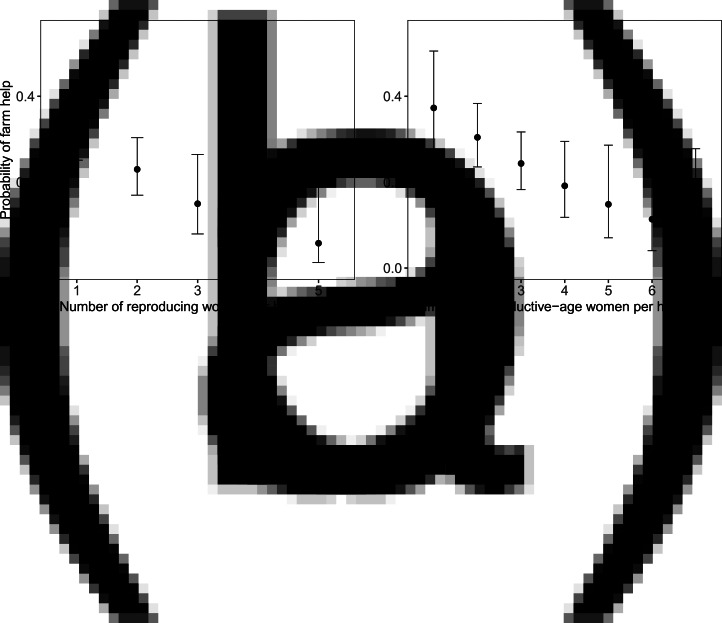
Predicted probability of women's household being helped by their spouses decreased as the number of (a) reproducing women, and (b) reproductive-age women per household increased. Error bars represent 95% CI.

Table 2 and Figure 4 show the results of the adjusted models for reproducing women in visiting marriages (*n* = 195), with whether the spouse was observed helping on one's farm or not (1= helped; 0 = not helped) as the dependent variable. A larger number of reproducing women and reproductive-age women in a reproducing woman's household predicted lower probability of her spouse helping her household (odds ratio = 0.59 and 0.74; 95% CI = [0.38, 0.92] and [0.55, 0.99], respectively, also see Figure 4), supporting prediction 2. That is, on average, when one more reproducing woman lives in a reproducing woman's household, the probability of her spouse helping on the farm of her household decreases about 41%, and one more reproductive-age woman reduces the probability by about 26%.

**Table 2.** Adjusted models for estimating the total effects of communal breeding on husband's help (*n* = 195 reproducing Mosuo women). The predictor of model 1 was number of reproducing women, and that of model 2 was the number of reproductive-age women. Models adjusted whether a woman is reproducing by only included reproducing women.

Variables	Model 1		Model 2	
	Odds ratio	95% CI	Odds ratio	95% CI
Intercept	0.85	[0.40, 1.84]	0.81	[0.36, 1.80]
Reproducing women	0.59	[0.38, 0.92]	—	—
Reproductive-age women	—	—	0.74	[0.55, 0.99]

### (c) Help of natal farms

**Figure 5.**
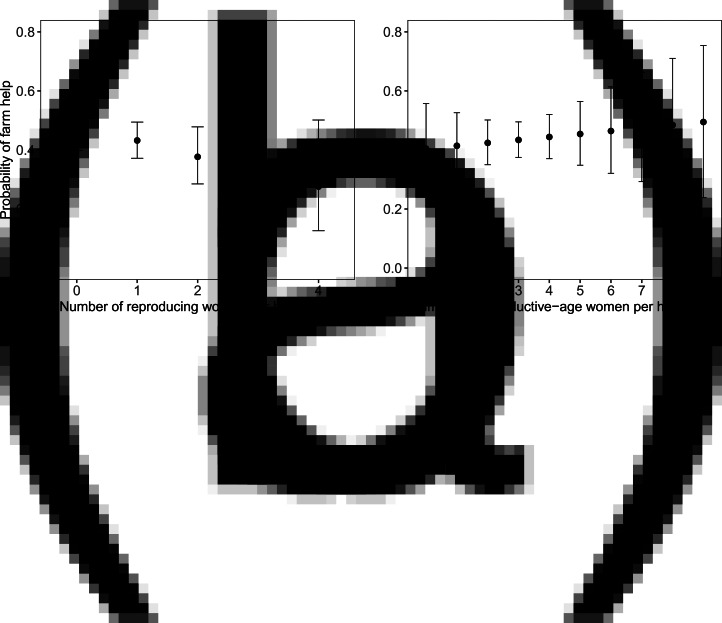
Predicted probability of a man helping his natal farm was not significantly correlated to the number of (a) reproducing women and (b) reproductive-age women per household. Error bars represent 95% CI.

Some 43.7% zouhun men helped their natal farms during the observation period (114 out of 261, Table S1). Unlike help on his wife's farm, high levels of communal breeding in a zouhun man's natal household did not significantly affect whether he worked on his natal farm (Table 3 and Figure 5). Neither the number of reproducing women (odds ratio = 0.79, 95% CI [0.59, 1.07]) nor that reproductive-age women (odds ratio = 1.04, 95% CI [0.86, 1.26]) significantly affected zouhun men helping on their natal farm, supporting prediction 3.

**Table 3.** Adjusted models for estimating the total effects of communal breeding on helping natal farms (*n* = 261 Mosuo men). The predictor of model 3 was the number of reproducing women, and that of model 4 was the number of reproductive-age women in a man's natal household. Both models adjusted for men's age.

Variables	Model 3		Model 4	
	Odds ratio	95% CI	Odds ratio	95% CI
Intercept	1.75	[0.67, 4.61]	1.32	[0.47, 3.71]
Age	0.99	[0.97, 1.01]	0.98	[0.96, 1.01]
Reproducing women	0.79	[0.59, 1.07]	—	—
Reproductive-age women	—	—	1.04	[0.86, 1.26]

## 4. Discussion

Our results show support for an important prediction of Wu et al.'s hypothesis (Wu et al., 2013) that high levels of communal breeding by women reduce men's help to their wives' households. In matrilineal societies, sisters and other relatives benefit from co-residing with each other, sharing resources and cooperating with child rearing (Leonetti et al., 2007; Sear & Mace, 2008). However, this decision might incur them some costs by decreasing investment from their husbands. We show that reduced investment from the husband was largely caused by diluted fitness return, rather than the asymptotic nature of the benefits of help on any one farm, as no effects of communal breeding on men's help on their sister's farm were found.

We had previously shown that matrilineal Mosuo were more likely to help on the farms of households where their sexual partners live (Thomas et al., 2018). Here we examine that effect of household structure in more detail. The number of reproductive-age women or reproducing women in a household significantly affects husbands' average relatedness to the household and the probability of men helping their wives' farm. Levels of communal breeding of a man's household did not affect his help on natal farms. These results indicate that decreases in a man's help on their wife's farm might be caused by fitness benefits being diluted by unrelated members of their wife's household, as Wu et al. (2013) predicted. This effect might have been even bigger in the past, when fewer adult women dispersed from their natal households, family sizes were bigger (He et al., 2016; Walsh, 2004) and culture norms about perceived fathers' responsibility were probably weaker (Xiao et al., 2022).

A man's efforts invested in his wife's farm could act as parental investment or/and mating effort (Andersson, 1994; Emlen & Oring, 1977; Marlowe, 1999a, 1999b; Trivers, 1972). Parental investment contributes to the wellbeing of a man's existing children. Mating effort helps him maintain a stable relationship with his wife and have more children with her in the future. Wu et al.'s (2013) hypothesis did not discriminate these two parts explicitly, but it mainly focused on paternal investment. Between-household cooperation patterns of the matrilineal Mosuo confirm that farm labour might act as a form of parental investment when children live with the helper's non-kin (Thomas et al., 2018). High levels of communal breeding would be expected to have little influence on the husband's allocation of mating effort or his fitness benefits derived from it. Our results of communal breeding in the wife's household reducing the husband's labour effort suggest that at least some of it acts as parental investment.

Women with dependents and reproductive-age women without dependents both compete for household resources (Ji et al., 2013). Therefore, this study used both the number of reproductive-age women and that of reproducing women to measure the level of communal breeding of households. Both variables were significantly associated with the husband's average relatedness to the household and the probability of husband's farm help, with the negative effects of reproducing women on both variables bigger than that of reproductive-age women. These results may indicate that an actual reduction in the husband's relatedness to the household when women are reproductive (and not simply reproductive-aged) drives a decrease in the husband's help.

In conclusion, we found that communal breeding by women was associated with lower man's investment in his wife's farm, which leaves time to increase his investment in his sister's farm. It is not entirely clear whether a man's saved time or labour efforts would be all invested in his sister's farms, but this is reasonable. Duolocal Mosuo men were more frequently seen on farms of their natal households than on their wives' farms (Wu et al., 2013). More importantly, it is highly possible that the levels of paternity uncertainty and efforts invested in enhancing extra-pair mating success would remain the same as the levels of communal breeding increase. If so, reduced efforts allocated to a man's wife would lead to an increase in investment in his sisters, although it should be noted that, given high levels of communal breeding of women, decreased paternal investment does not necessarily harm a man's children's wellbeing (Mattison et al., 2019). Furthermore, other forms of investment, such as direct care or resources that are more easily monopolised, might be less likely to be influenced by communal breeding of women in the wife's household than is the labour effort.

Our results should also apply to other matrilineal societies with matrilocal residence. Communal breeding and harvest sharing of women appear to have been rather common in matrilineal groups (Ember, 1971; Peletz, 1988). Thus, it is likely that communal breeding by related women in matrilineal groups drives the evolution of a man's matrilineal investment (rather than investing in his wife and offspring), thus facilitating the maintenance of the matrilineal systems and helping to solve the ‘matrilineal puzzle'.

## Research transparency and reproducibility

Data supporting the findings in this study are not publicly available due to the need to preserve anonymity. Code used to run our GEE models and figures were included in a R Markdown document as electronic supplementary material.

## Supplementary material

For supplementary material accompanying this paper visit https://doi.org/10.1017/ehs.2022.47. click here to view supplementary material
