# Communal breeding by women is associated with lower investment from husbands – ERRATUM

**DOI:** 10.1017/ehs.2022.56

**Published:** 2022-12-13

**Authors:** Qiao-Qiao He, Jun-Wen Rui, Li Zhang, Yi Tao, Jia-Jia Wu, Ruth Mace, Ting Ji

The publisher apologises that upon publication of He Q-Q, Rui J-W, Zhang L, Tao Y, Wu J-J, Mace R, Ji T (2022) the affiliation for author Ting Ji was incorrectly listed and they were not noted as a corresponding author. The correct affiliations are listed as below:

Qiao-Qiao He^1^, Jun-Wen Rui^1^, Li Zhang^1^, Yi Tao^2^, Jia-Jia Wu^3^, Ruth Mace^4^ and Ting Ji^2^

^1^College of Life Science, Shenyang Normal University, Shenyang, Liaoning 110034, China,

^2^Key Laboratory of Animal Ecology and Conservation Biology, Institute of Zoology, Chinese Academy of Sciences, Beijing 100101, China,

^3^Life Science, Lanzhou University, Tianshui Rd, Chengguan Qu, Lanzhou, Gansu Province, 730000, China and

^4^Department of Anthropology, University College London, London WC1H 0BW, UK

The correct corresponding author list is listed as below:

Corresponding author: r.mace@ucl.ac.uk, jiting@ioz.ac.cn

The online version of this article has been updated.

## References

[ref1] He, Q-Q., Rui, J-W., Zhang, L., Tao, Y., Wu, J-J., Mace, R., Ji, T. (2022). Communal breeding by women is associated with lower investment from husbands. Evolutionary Human Sciences 4, e50, 1–12. 10.1017/ehs.2022.47 PMC10426074 37588900

