# Supplementary material for: Communal breeding by women is associated with lower investment from husbands
Source: Evol Hum Sci. 2022 Oct 20;4:e50. doi: 10.1017/ehs.2022.47 (PMC10426074; doi:10.1017/ehs.2022.47)

Figures in Communal breeding by women is associated with lower investment from husbands

Qiao-Qiao He, Jun-Wen Rui, Li Zhang, Yi Tao, Jia-Jia Wu, Ruth Mace, Ting Ji

2022-10-07

## Introduction

This R Markdown document was generated using RStudio. It shows the code of our gee models and figures 2-5.

## Packages used

library(tidyverse)
library(wesanderson)
library(dplyr)
library(ggplot2)
library(geepack)
library(gridExtra)
library(aod)
library(data.table)
library(geepack)

## Figure 2

##fig 2

##
## plot correlations between predictors
##
# get upper triangle of the correlation matrix
# (source: http://www.sthda.com/english/wiki/ggplot2-quick-correlation-matrix-heatmap-r-software-and-data-visualization) see Thomas et al 2016
get_upper_tri <- function(cormat){
 cormat[lower.tri(cormat)]<- NA
 return(cormat)
}


d = FarmVM1msb %>%
 select(PhelpedMe,breedingF0712, BF, PChdinHH, pAvgR,Age )

cor_vars = c("Husband helped?","Reproducing women","Reproductive-age women","Husband's children",
 "Relatedness", "Age")


# calculate correlations and p-values
cormats = d %>%
 as.matrix(.) %>%
 Hmisc::rcorr(.,type = "spearman")

# format p-values
cormat.p = cormats$P %>%
 round(., 2) %>%
 get_upper_tri(.) %>%
 reshape2::melt(.)

# format correlations and merge with p-values
cormat = cormats$r %>%
 round(., 2) %>%
 get_upper_tri(.) %>%
 reshape2::melt(.) %>%
 rename(r = value) %>%
 left_join(cormat.p, by=c("Var1", "Var2")) %>%
 rename(p = value)

# rename variables
cormat$Var1 = factor(cormat$Var1)
levels(cormat$Var1) = cor_vars
cormat$Var2 = factor(cormat$Var2)
levels(cormat$Var2) = cor_vars


ggplot(data = cormat, aes(Var2, Var1, fill = p)) +
 geom_tile(colour = "white") +
 geom_text(aes(label = r), size=3) +
 scale_fill_gradient2(low = "red", high = "blue", mid = "white", na.value="grey75",
 midpoint = 0.05, limit = c(0, 0.1), space = "Lab",
 name="P-value") +

 theme_minimal() +
 theme(axis.text.x = element_text(angle = 45, vjust = 1,
 size = 11, hjust = 1)) +
 coord_fixed() +
 xlab("") +
 ylab("")

# point out where p < 0.01 (rather than show it as p = 0)
cormat$p = as.character(cormat$p)
cormat$p = ifelse(cormat$p == "0", "< 0.01", cormat$p)
cormat

## Warning: Removed 15 rows containing missing values (geom_text).


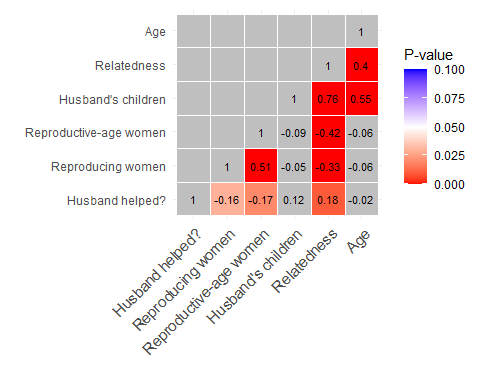


## Figure 3

#data explore and fig 3

#wilcox test
wilcox.test(FarmVM1msb$BF~FarmVM1msb$PhelpedMe)
wilcox.test(FarmVM1msb$breedingF0712~FarmVM1msb$PhelpedMe)
wilcox.test(FarmVM1msb$pAvgR~FarmVM1msb$PhelpedMe)
wilcox.test(FarmVM1msb$BFPartner~FarmVM1msb$PhelpedMe)


#fig 3
# breeding female (not reproductive-age women)
opar <- par(no.readonly=TRUE)
par(mfrow=c(1,3))
boxplot (FarmVM1msb$BF ~ FarmVM1msb$PhelpedMe, title= "",
 #ylim = c(0, 8),
 ylab = "Reproductive-age women in my household",
 xlab = "Husband helped my household")
boxplot (FarmVM1msb$breedingF0712 ~ FarmVM1msb$PhelpedMe,
 #ylim = c(0, 5),
 ylab = "Breeding females in my household",
 xlab = "Husband helped my household")

boxplot (FarmVM1msb$pAvgR ~ FarmVM1msb$PhelpedMe,
 #ylim = c(0, 0.5),
 ylab = "Average relatedness of spouse to my household",
 xlab = "Husband helped my household")
par(opar)


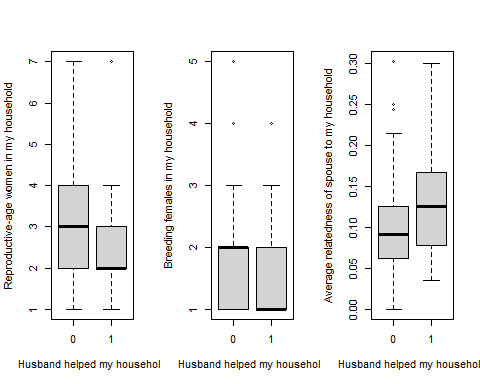


## GEE models and Figures 4-5

####men help wife's household

###GEE


Farmgee.sig<-geeglm (PhelpedMe ~breedingF0712#reproducing women
 ,id=HH,
 family="binomial",
 data =subset(FarmVM1msb), #only reproducing women (with dependant) included
 corstr="exchangeable", scale.fix=T)
summary (Farmgee.sig)
exp(coef(Farmgee.sig, full=T))
Farmgee.sigraw<-geeglm (PhelpedMe ~BF #reproducitve-age women
 ,id=HH,
 family="binomial",
 data = subset(FarmVM1msb), #only reproducing women (with dependant) included
 corstr="exchangeable", scale.fix=T)
summary (Farmgee.sigraw)
exp(coef(Farmgee.sigraw, full=T))

####fig 4
############################################################################
## Function to calculate 95% CIs for GEE models
## adapted from: https://danieljhocking.wordpress.com/2012/07/25/plotting-95-confidence-bands-in-r/ (see Thomas et al. 2016)
##
m.terms = terms(Farmgee.sig)

predict_gee = function(m, m.terms, new.data)
{
 mm.geeEX = model.matrix(m.terms, new.data) # use the full model to create the design matrix, since can't run terms() on an averaged model
 new.data$PhelpedMe = mm.geeEX %*% coef(m)

 tvar1.gee = diag(mm.geeEX %*% tcrossprod(aod::vcov(m), mm.geeEX))

 new.data = data.frame(
 new.data
 , tlo = new.data$PhelpedMe - 2*sqrt(tvar1.gee)
 , thi = new.data$PhelpedMe + 2*sqrt(tvar1.gee)
 )

 # keep log odds
 # new.data$PhelpedMe_logodds = new.data$PhelpedMe
 # new.data$tlo_logodds = new.data$tlo
 # new.data$thi_logodds = new.data$thi

 # transform log odds to probabilities
 new.data$PhelpedMe = as.numeric(plogis(new.data$PhelpedMe))
 new.data$tlo = plogis(new.data$tlo)
 new.data$thi = plogis(new.data$thi)
 return(new.data)
}

##
## to make the plots look pretty
##
plot_style = theme_bw() +
 #eliminates baground, gridlines, and chart border
 theme(
 plot.background = element_blank()
 ,panel.grid.major = element_blank()
 ,panel.grid.minor = element_blank()
 ,panel.background = element_blank()
 ,axis.text=element_text(size=12)
 ,axis.title=element_text(size=12)
 ) +
 theme(legend.position="bottom", legend.title=element_blank()) # legends at the bottom, with no titles


## reproducing women
##
library(aod)
new.data = expand.grid(breedingF0712=seq(from=1, to=5, length.out = 5),
 breedingF0712.c = c(0, mean(FarmVM1msb$breedingF0712), max(FarmVM1msb$breedingF0712)),
 # set these predictors to their means
 PhelpedMe=0)

new.data = predict_gee(Farmgee.sig, m.terms, new.data)

new.data$breedingF0712.c = factor(new.data$breedingF0712.c)
levels(new.data$breedingF0712.c) = c("None", "Mean", "Five")

plt.breedingf = new.data %>%
 #filter(Ego.VillageID==1) %>%
 ggplot(aes(x=as.factor(breedingF0712), y=PhelpedMe)) +
 geom_point(size=2) +
 geom_errorbar(aes(ymax=thi, ymin=tlo), width=.2, size=.3) +
 xlab("Number of reproducing women per household") +
 ylab("Probability of farm help") +
 ylim(0,0.55)+
 plot_style
plt.breedingf

###reproductive-age women
############################################################################
## Function to calculate 95% CIs for GEE models
## adapted from: https://danieljhocking.wordpress.com/2012/07/25/plotting-95-confidence-bands-in-r/
##
m.terms1 = terms(Farmgee.sigraw)

predict_gee = function(m1, m.terms1, new.data1)
{
 mm.geeEX1 = model.matrix(m.terms1, new.data1) # use the full model to create the design matrix, since can't run terms() on an averaged model
 new.data1$PhelpedMe = mm.geeEX1 %*% coef(m1)

 tvar1.gee1 = diag(mm.geeEX1 %*% tcrossprod(aod::vcov(m1), mm.geeEX1))

 new.data1 = data.frame(
 new.data1
 , tlo = new.data1$PhelpedMe - 2*sqrt(tvar1.gee1)
 , thi = new.data1$PhelpedMe + 2*sqrt(tvar1.gee1)
 )

 new.data1$PhelpedMe = as.numeric(plogis(new.data1$PhelpedMe))
 new.data1$tlo = plogis(new.data1$tlo)
 new.data1$thi = plogis(new.data1$thi)
 return(new.data1)
}

new.data1 = expand.grid(BF=seq(from=1, to=7, length.out = 7),
 BF.c = c(0, mean(FarmVM1msb$BF), max(FarmVM1msb$BF)),
 # set these predictors to their means
 PhelpedMe=0)
 # set response to zero for now

new.data1 = predict_gee(Farmgee.sigraw, m.terms1, new.data1)


plt.RAW = new.data1 %>%
 ggplot(aes(x=as.factor(BF), y=PhelpedMe)) +
 geom_point(size=2) +
 geom_errorbar(aes(ymax=thi, ymin=tlo), width=.2, size=.3) +
 xlab("Number of reproductive-age women per household") +
 ylab("") +
 ylim(0,0.55)+
 plot_style
plt.RAW

##
## Save the plots
##
# first give them titles
plt.breedingf = plt.breedingf + ggtitle("A")
plt.RAW = plt.RAW + ggtitle("B")


png(filename = file.path( "fig 4.png"), height=10, width=22.5, units = "cm", res=300) #all women
grid.arrange(plt.breedingf, plt.RAW,
 ncol=2, nrow=1)
dev.off()

pdf(file = file.path( "fig 4.pdf"), height=4, width=9) #all women
grid.arrange(plt.breedingf, plt.RAW,
 ncol=2, nrow=1)
dev.off()


#########################################################################################
#men help natal
duomosuo0_ownerH<-read.table("C:/Users/heqq/Documents/duomosuo0_ownerH.csv",sep = ",",header = TRUE)
###GEE

Farm_DML3.11 <- geeglm (AsOwner ~ Age+
 RAW
 ,id=MC.H2012,
 family="binomial", corstr="exchangeable", scale.fix=T,
 data = subset(duomosuo0_ownerH, !is.na(RAW)))
summary (Farm_DML3.11)
exp(coef(Farm_DML3.11, full=T))


Farm_DML4.11 <- geeglm (AsOwner ~ Age+
 BW
 ,id=MC.H2012,
 family="binomial", corstr="exchangeable", scale.fix=T,
 data = subset(duomosuo0_ownerH, !is.na(BW)))
summary (Farm_DML4.11)
exp(coef(Farm_DML4.11, full=T))


##fig 5
############################################################################
## Function to calculate 95% CIs for GEE models
## adapted from: https://danieljhocking.wordpress.com/2012/07/25/plotting-95-confidence-bands-in-r/
##
m.terms = terms(Farm_DML4.11)

predict_gee = function(m, m.terms, new.data)
{
 mm.geeEX = model.matrix(m.terms, new.data) # use the full model to create the design matrix, since can't run terms() on an averaged model
 new.data$AsOwner = mm.geeEX %*% coef(m)

 tvar1.gee = diag(mm.geeEX %*% tcrossprod(aod::vcov(m), mm.geeEX))

 new.data = data.frame(
 new.data
 , tlo = new.data$AsOwner - 2*sqrt(tvar1.gee)
 , thi = new.data$AsOwner + 2*sqrt(tvar1.gee)
 )


 new.data$AsOwner = as.numeric(plogis(new.data$AsOwner))
 new.data$tlo = plogis(new.data$tlo)
 new.data$thi = plogis(new.data$thi)
 return(new.data)
}

##
## to make the plots look pretty
##
plot_style = theme_bw() +
 #eliminates baground, gridlines, and chart border
 theme(
 plot.background = element_blank()
 ,panel.grid.major = element_blank()
 ,panel.grid.minor = element_blank()
 ,panel.background = element_blank()
 ,axis.text=element_text(size=12)
 ,axis.title=element_text(size=12)
 ) +
 theme(legend.position="bottom", legend.title=element_blank()) # legends at the bottom, with no titles


## reproducing women
##
library(aod)
new.data = expand.grid(BW=seq(from=0, to=4, length.out = 5),
 BW.c = c(0, mean(duomosuo0_ownerH$BW), max(duomosuo0_ownerH$BW)),
 # set these predictors to their means
 Age =mean(duomosuo0_ownerH$Age),
 # set response to zero for now
 AsOwner=0)

new.data = predict_gee(Farm_DML4.11, m.terms, new.data)

#new.data$BW.c = factor(new.data$BW.c)
#levels(new.data$BW.c) = c("None", "Mean", "Five")

plt.breedingf1 = new.data %>%
 #filter(Ego.VillageID==1) %>%
 ggplot(aes(x=as.factor(BW), y=AsOwner)) +
 geom_point(size=2) +
 geom_errorbar(aes(ymax=thi, ymin=tlo), width=.2, size=.3) +
 xlab("Number of reproducing women per household") +
 ylab("Probability of farm help") +
 ylim(0,0.8)+
 plot_style

plt.breedingf1

###reproductive-age women
############################################################################
## Function to calculate 95% CIs for GEE models
## adapted from: https://danieljhocking.wordpress.com/2012/07/25/plotting-95-confidence-bands-in-r/
##
m.terms1 = terms(Farm_DML3.11)

predict_gee = function(m1, m.terms1, new.data1)
{
 mm.geeEX1 = model.matrix(m.terms1, new.data1) # use the full model to create the design matrix, since can't run terms() on an averaged model
 new.data1$AsOwner = mm.geeEX1 %*% coef(m1)

 tvar1.gee1 = diag(mm.geeEX1 %*% tcrossprod(aod::vcov(m1), mm.geeEX1))

 new.data1 = data.frame(
 new.data1
 , tlo = new.data1$AsOwner - 2*sqrt(tvar1.gee1)
 , thi = new.data1$AsOwner + 2*sqrt(tvar1.gee1)
 )

 new.data1$AsOwner = as.numeric(plogis(new.data1$AsOwner))
 new.data1$tlo = plogis(new.data1$tlo)
 new.data1$thi = plogis(new.data1$thi)
 return(new.data1)
}
library(aod)
new.data1 = expand.grid(RAW=seq(from=0, to=9, length.out = 10),
 RAW.c = c(0, mean(duomosuo0_ownerH$RAW), max(duomosuo0_ownerH$RAW)),
 # set these predictors to their means
 Age =mean(duomosuo0_ownerH$Age),
 # set response to zero for now
 AsOwner=0)

new.data1 = predict_gee(Farm_DML3.11, m.terms1, new.data1)


plt.RAW1 = new.data1 %>%
 ggplot(aes(x=as.factor(RAW), y=AsOwner)) +
 geom_point(size=2) +
 geom_errorbar(aes(ymax=thi, ymin=tlo), width=.2, size=.3) +
 xlab("Number of reproductive-age women per household") +
 ylab("") +
 ylim(0,0.8)+
 plot_style
plt.RAW1

##
## Save the plots
##
# first give them titles
plt.breedingf1 = plt.breedingf1 + ggtitle("A")
plt.RAW1 = plt.RAW1 + ggtitle("B")


png(filename = file.path( "fig 5.png"), height=10, width=22.5, units = "cm", res=300)
grid.arrange(plt.breedingf1, plt.RAW1,
 ncol=2, nrow=1)
dev.off()

pdf(file = file.path( "fig 5.pdf"), height=4, width=9)
grid.arrange(plt.breedingf1, plt.RAW1,
 ncol=2, nrow=1)
dev.off()

### Figure 4


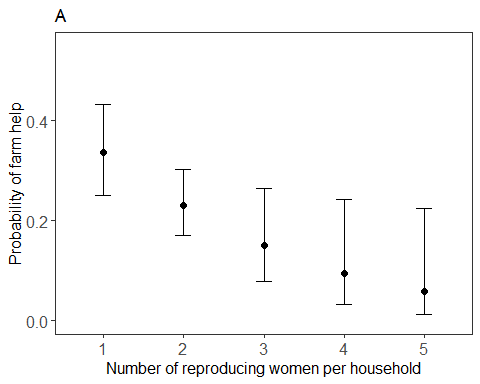

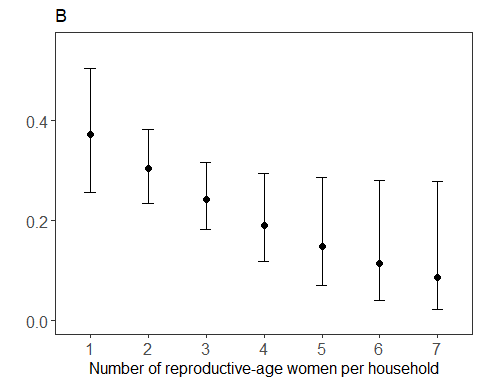


### Figure 5


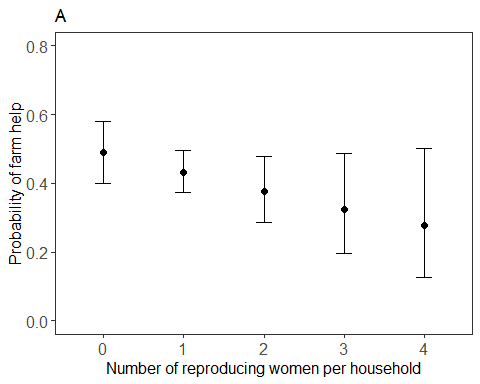

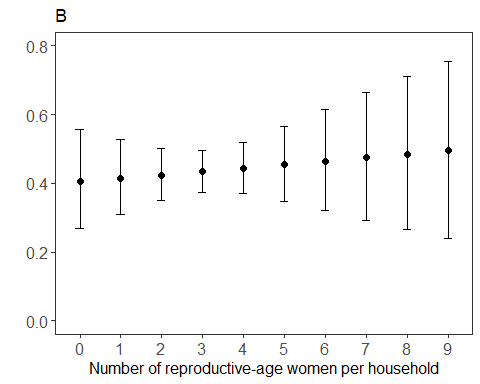

Supplement: Supplementary file 1 [file S2513843X22000470sup.zip › S2513843X22000470sup002.docx]
