# Supplementary material for: Communal breeding by women is associated with lower investment from husbands
Source: Evol Hum Sci. 2022 Oct 20;4:e50. doi: 10.1017/ehs.2022.47 (PMC10426074; doi:10.1017/ehs.2022.47)
Supplement: Supplementary file 1 [file S2513843X22000470sup.zip › S2513843X22000470sup001.docx]

**Supplementary material of**

**Communal breeding by women is associated with lower investment from husbands**

Qiao-Qiao He ^a^, Jun-Wen Rui ^a^, Li Zhang ^a^, Yi Tao ^b^, Jia-Jia Wu ^c^, Ruth Mace ^d^*, Ting Ji ^b^*

^a^ College of Life Science, Shenyang Normal University, Shenyang, Liaoning 110034, China

^b^ Key Laboratory of Animal Ecology and Conservation Biology, Institute of Zoology, Chinese Academy of Sciences, Beijing 100101, China

^c^ Life Science, Lanzhou University, Tianshui Rd, Chengguan Qu, Lanzhou, Gansu Province, 730000, China

^d^ Department of Anthropology, University College London, London WC1H 0BW, UK

* Corresponding authors.

E-mail addresses: jiting@ioz.ac.cn, r.mace@ucl.ac.uk

**S1 Directed acyclic graphs**

**S1.1 Help on wife’s farm**

We used directed acyclic graphs (DAGs) to analyse the causal effect of communal breeding by women (exposure) on whether a woman’s husband helped her farm (outcome) (Pearl, 2000). We illustrated a directed acyclic graph with number of breeding/reproductive-age women in wife’s household as exposure (reproducing women in mine, Figure 1a). A man’s fitness benefits from investment in his wife’s household are determined by his average relatedness to his wife’s household (Wu et al., 2013). In this DAG, husband’s average relatedness to a woman’s household acts as a mediator of the causal effect of levels of communal breeding by women in a woman’s household on husband’s help on her farm (Pearl, 2000). We also considered all the other possible confounders (Figure 1a) (Thomas et al., 2018; Wu et al., 2013), and selected the minimum sufficient adjustment sets (Pearl, 2000; Textor & Hardt, 2011; Textor & Liśkiewicz, 2011) for estimating the total effects of communal breeding of women on whether a woman’s husband helped her farm.

A man’s effort could be invested in three possible domains, his sister, wife or extra-pair mating (Wu et al., 2013). The amount of effort invested in extra-pair mating is mainly determined by paternity certainty (Wu et al., 2013), and both of them were unobserved variables in the DAG (Figure 1a). Spatial distance between households is a constraint of cooperation, especially for non-kin (Thomas et al., 2018). Besides, a man may be less confident about paternity certainty if his wife lives far away. As the age of a woman increases, we expected that more children (of any age) fathered by her husband and higher average relatedness between husband and her household. In addition, a woman of certain age is more likely to have dependants living with her (defined as “reproducing”). Husbands of elder women are less likely to help on their farms. According to Wu et al. (2013), we restricted our analyses to Mosuo zouhun women with dependants aged 14 or less (reproducing women).

In addition, we found no evidence against conditional independence between number of reproducing/reproductive-age women and husband’s help given husband’s average relatedness--husband’s average relatedness acts as a mediator (Pearl, 2000). Consistent of DAGs and data was analysed with localTests function in “dagitty” package (Textor et al., 2016). DAG-dataset consistency evaluation supported the conditional independence between number of reproducing (loess regression, estimate = -0.075, 95% CI [-0.186, 0.078]) or reproductive-age women (loess regression, estimate = -0.093, 95% CI [-0.192, 0.017]) in a reproducing woman’s household and whether her husband helped or not, given age and her husband’s average relatedness to her household. Note that these tests were based on an incomplete DAG to allow the analyses, with unobserved variables (paternity certainty and husband’s investment to extra-pair mating) or variables of constant values (reproducing or not) in the dataset excluded. Moreover, only complete cases were included in the analyses.

**S1.2 Help on natal farm**

We also used directed acyclic graphs (DAGs) to analyse whether there was any effect of communal breeding by women (exposure) on whether a man helped his natal farms (outcome) (Pearl, 2000). We illustrated a directed acyclic graph with number of breeding/reproductive-age women in a man’s household as exposure (reproducing women in natal, Figure 1b). We also considered all the other possible confounders (Figure 1b) (Thomas et al., 2018; Wu et al., 2013), and selected the minimum sufficient adjustment set (Pearl, 2000; Textor & Hardt, 2011; Textor & Liśkiewicz, 2011) for estimating the total effects of communal breeding of women on whether a man helped his natal farm. Note that a man’s average relatedness to his natal household would not be affected by communal breeding of female kin much (represented by no link from reproducing women in natal to r to natal).

**S2 Descriptive statistics of 261 zouhun men**

**Table S1 Descriptive statistics of 334 Mosuo women and their households.**

|  | Households | Men |
| --- | --- | --- |
| Zouhun men | 172 | 261 |
| Mean age ± SD (range) |  | 43.39 ± 12.07 (16-88) |
| Number of men ever helped natal | 92 | 114 |
| Number of men never helped natal | 117 | 147 |
| Mean number of breeding women per household ± SD (range) |  | 0.94 ± 0.86 (0-4) |
| Ever helped |  | 0.84 ± 0.81 (0-4) |
| Never helped |  | 1.01 ± 0.89 (0-4) |
| Mean number of reproductive-age women per household ± SD (range) |  | 3.22 ± 1.49 (0-9) |
| Ever helped |  | 3.25 ± 1.51 (0-9) |
| Never helped |  | 3.19 ± 1.47 (0-7) |
| Average relatedness |  | 0.33± 0.11 (0-1) |
| Ever helped |  | 0.34 ± 0.11 (0.113-1) |
| Never helped |  | 0.32 ± 0.11 (0-0.50) |

**References**

Alexander, R. D. (1974). The evolution of social behavior. *Annual Review of Ecology and Systematics*, *5*(1), 325–383. https://doi.org/10.1146/annurev.es.05.110174.001545

Anderson, K. G. (2006). How well does paternity confidence match actual paternity? Evidence from worldwide nonpaternity rates. *Current Anthropology*, *47*(3), 513–520. https://doi.org/10.1086/504167

Greene, P. J. (1978). Promiscuity, paternity, and culture. *American Ethnologist*, *5*(1), 151–159. https://doi.org/10.1525/ae.1978.5.1.02a00110

Pearl, J. (2000). *Causality: Models, reasoning, and inference*. Cambridge University Press.

Textor, J., & Hardt, J. (2011). DAGitty: A graphical tool for analyzing causal diagrams. *Epidemiology*, *22*(5), 745. https://doi.org/10.1097/EDE.0b013e318225c2be

Textor, J., & Liśkiewicz, M. (2011). Adjustment criteria in causal diagrams: An algorithmic perspective. *Proceedings of the 27th Conference on Uncertainty in Artificial Intelligence, UAI 2011*, 681–688.

Textor, J., van der Zander, B., Gilthorpe, M. S., Liśkiewicz, M., & Ellison, G. T. (2016). Robust causal inference using directed acyclic graphs: The R package “dagitty.” *International Journal of Epidemiology*, *45*(6), 1887–1894. https://doi.org/10.1093/ije/dyw341

Thomas, M. G., Ji, T., Wu, J.-J., He, Q.-Q., Tao, Y., & Mace, R. (2018). Kinship underlies costly cooperation in Mosuo villages. *Royal Society Open Science*, *5*(2), 171535. https://doi.org/10.1098/rsos.171535

Trivers, R. (1972). Parental investment and sexual selection. In Campbell B (Ed.), *Sexual Selection and the Descent of Man: 1871-1971* (pp. 136–179). Aldine-Atherton. http://www.mendeley.com/research/parental-investment-and-sexual-selection/

Wu, J. J., He, Q.-Q. Q., Deng, L.-L. L., Wang, S. C., Mace, R., Ji, T., & Tao, Y. (2013). Communal breeding promotes a matrilineal social system where husband and wife live apart. *Proceedings of the Royal Society B: Biological Sciences*, *280*(1758), 20130010. https://doi.org/10.1098/rspb.2013.0010
